# Risk factors and prognosis of hypoalbuminemia in surgical septic patients

**DOI:** 10.7717/peerj.1267

**Published:** 2015-10-01

**Authors:** Jia-Kui Sun, Fang Sun, Xiang Wang, Shou-Tao Yuan, Shu-Yun Zheng, Xin-Wei Mu

**Affiliations:** Department of Intensive Care Unit, Nanjing First Hospital, Nanjing Medical University, Nanjing, Jiangsu Province, China

**Keywords:** Hypoalbuminemia, Sepsis, Septic shock, Risk factor, Prognosis

## Abstract

The aim of this study was to investigate the risk factors of hypoalbuminemia and effects of different albumin levels on the prognosis of surgical septic patients. We preformed a retrospective clinical study including 135 adult patients from September 2011 to June 2014. The albumin levels and severity markers were recorded during the first 48 h after enrollment, and logistic regression analyses were used to determine the risk factors. The outcomes of patients with different albumin levels were also compared. The acute physiology and chronic health evaluation II (APACHE II) score (OR 1.786, 95% CI [1.379–2.314], *P* < 0.001), C-reactive protein (CRP) (OR 1.016, 95% CI [1.005–1.027], *P* = 0.005), and blood lactate (OR 1.764, 95% CI [1.141–2.726], *P* = 0.011) were established as the independent risk factors of hypoalbuminemia in patients with surgical sepsis. The severity markers and outcomes of patients with albumin levels ≤20 g/L were significantly worse than that of 21–25 g/L and ≥26 g/L, whereas the latter two groups had similar prognosis. Every 1 g/L decrease of albumin level below the optimal cut-off (23 g/L) was associated with a 19.4% increase in hospital mortality and a 28.7% increase in the incidence of multiple organ dysfunction syndrome. In conclusion, APACHE II score (≥14.5), CRP (≥34.25 mg/L), and blood lactate (≥.35 mmol/L) were established as the independent risk factors of hypoalbuminemia in the early stage of surgical sepsis. Patients with baseline albumin level ≤20 g/L had worse prognosis than that of albumin level ≥21 g/L. Albumin levels were negatively correlated the prognosis of surgical sepsis when below about 23 g/L.

## Introduction

Sepsis is a systemic host response to infection, a small proportion of which may progress to severe sepsis and septic shock (Caironi et al., 2014; Dellinger et al., 2013). Severe sepsis and septic shock are extremely important causes of morbidity and mortality in intensive care units (ICUs) (Caironi et al., 2014; Patel et al., 2014). Despite the latest surviving sepsis guidelines have suggested a series of therapeutic principles of this fatal disease (Dellinger et al., 2013; Levy et al., 2010), its mortality in adults is still as high as 24–50% (Dellinger et al., 2013; Patel et al., 2014). Sepsis can induce hypoalbuminemia due to several pathophysiological mechanisms, and can also exacerbate the severity of sepsis (Aguayo-Becerra et al., 2013; Gatta, Verardo & Bolognesi, 2012). Nevertheless, the current researches mainly focus on using albumin as one kind of colloids for fluid resuscitation in patients with severe sepsis and septic shock. Controversially, a recent multicenter, open-label trial observed that albumin replacement in addition to crystalloids did not improve the survival rate of patients with severe sepsis (Caironi et al., 2014). The above findings indicate that albumin is not just used for resuscitation, but has special attributes and functions.

As a matter of fact, previous studies revealed that low albumin was an independent risk factor and an indicator of mortality in critical ill patients (Aguayo-Becerra et al., 2013; Chou et al., 2009; Gatta, Verardo & Bolognesi, 2012; SAFE Study Investigators et al., 2011). Aguayo-Becerra et al. (2013) reported that low albumin level (<2 g/dL) was a risk factor for mortality (risk >80%) in burn patients. Qian & Liu (2012) also found that serum albumin level was closely related to the prognosis of children with sepsis, severe sepsis or septic shock. However, it is still unclear whether the mortality is definitively dependent on albumin concentration (Patel et al., 2014). In addition there are rare studies investigating the effects of different albumin levels on the disease severity and outcomes for adults with sepsis, especially surgical sepsis. Moreover, there is also a lack of published articles with respect to risk factors of hypoalbuminemia in patients with sepsis. Therefore, in view of our surgical ICU (SICU) population (mostly surgical sepsis), the present retrospective study aimed to investigate the risk factors for developing hypoalbuminemia, as well as the effects of different albumin levels on the prognosis of surgical septic patients.

## Materials and Methods

### Patients

**Figure 1:**
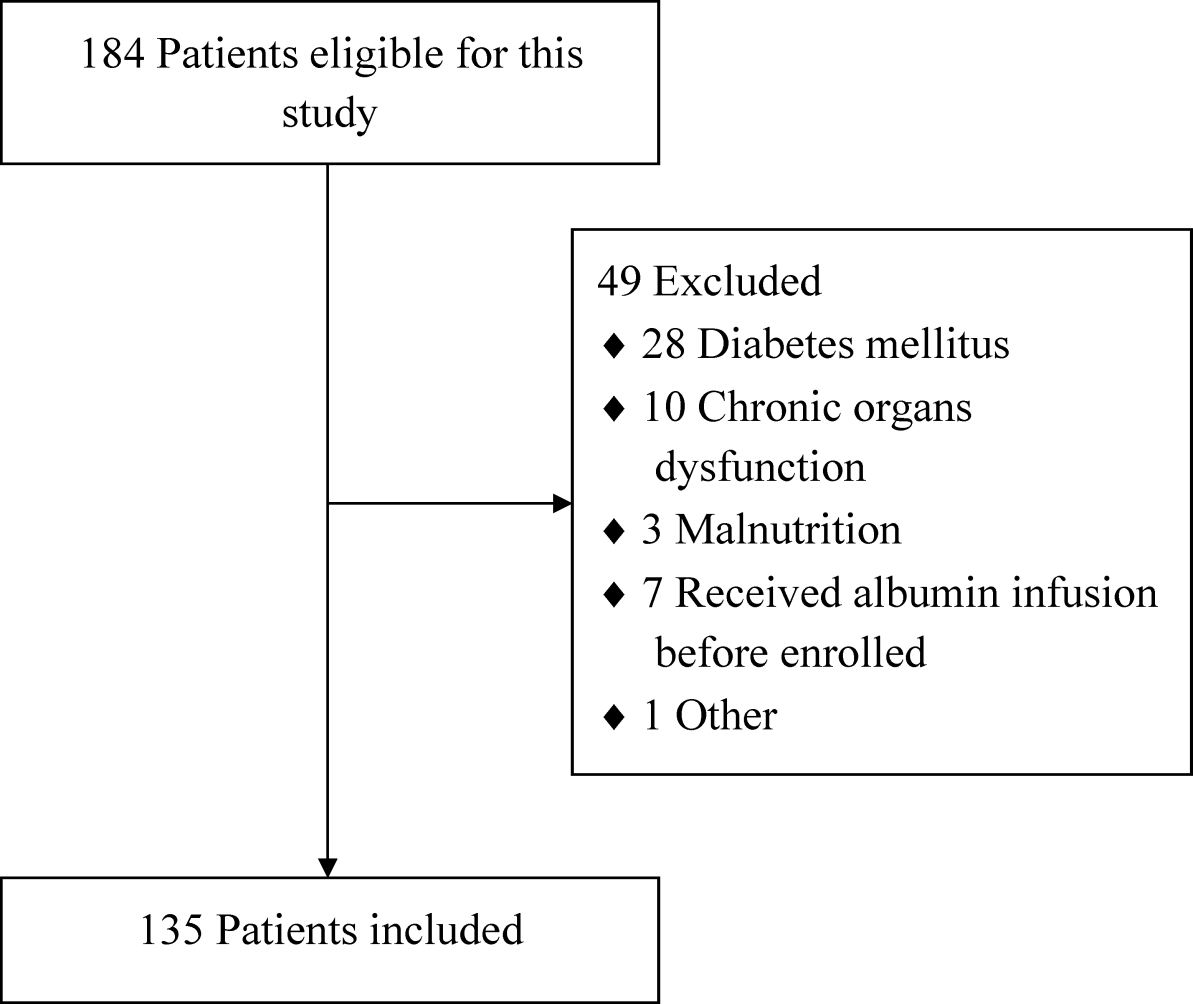
The flow diagram of participants.

From September 2011 to June 2014, all consecutive adult patients admitted into our department of SICU, Nanjing First Hospital, with sepsis and ICU stay of 48 h or more, were included in this study. The diagnostic criterion of sepsis was accordant with the surviving sepsis guidelines (Dellinger et al., 2008; Dellinger et al., 2013). Patients with chronic organ dysfunction (e.g., hepatic or renal dysfunction), coagulation dysfunction, diabetes mellitus, malnutrition or immunodeficiency, and patients who had received albumin infusion before admission were all excluded. Figure 1 showed the flow diagram of participants. The study was approved by the institutional review board of the hospital, and written informed consent was waived because this was a retrospective study. All patients received specialized treatment for sepsis such as intensive monitoring, fluid resuscitation, oxygen administration or mechanical ventilation (MV), antimicrobial therapy, vasopressor administration, glucose control, nutrition support, renal replacement therapy and so on (Dellinger et al., 2008; Dellinger et al., 2013; Levy et al., 2010).

### Definitions

Sepsis was defined as the presence of infection together with systemic inflammatory response syndrome (SIRS, diagnosed when two or more of the following criteria are met: body temperature <36 °C or >38 °C; heart rate >90 beats/min; tachypnea >20 breaths/min, or an arterial partial pressure of carbon dioxide <32 mmHg; white blood cell count less than 4 × 10^9^/L or greater than 12 × 10^9^/L, or the presence of >10% immature neutrophils). Severe sepsis was diagnosed as sepsis plus sepsis-induced organ dysfunction or tissue hypoperfusion. Septic shock was defined as severe sepsis plus hypotension not reversed by fluid resuscitation (Dellinger et al., 2008; Dellinger et al., 2013). Hypoalbuminemia is generally defined by a serum albumin <35 g/L, however, considering the clinical circumstances of sepsis, we defined hypoalbuminemia as an albumin level of less than 25 g/L in this study. The diagnostic criteria of acute respiratory distress syndrome (ARDS) were accordant with the Berlin definitions (ARDS Definition Task Force et al., 2012): (1) the onset must be within one week of respiratory symptoms; (2) chest imaging showed pulmonary bilateral opacities; (3) emerging respiratory failure not fully explained by cardiac failure or fluid overload; and (4) PaO2/FIO2 ≤ 300 mmHg with positive end-expiratory pressure (PEEP) ≥5 cm H2O. The definitions of acute kidney injury (AKI) were based on the Kidney Disease: Improving Global Outcomes (KDIGO) guidelines in 2012 (Kellum, Lameire & KDIGO AKI Guideline Work Group , 2013). AKI was defined as any of the followings: serum creatinine increased to 1.5 or more times baseline or increased by ≥26.5 µmol/L within 48 h; or urine volume <0.5 ml/kg/h for 6 h. Intra-abdominal hypertension (IAH) was defined by a sustained or repeated pathological elevation in intra-abdominal pressure (IAP) ≥12 mmHg. Multiple organ dysfunction syndrome (MODS) was defined as the combined dysfunction of two or more organs.

### Data collection

On admission, the baseline characteristics including age, sex, body mass index (BMI), and etiology of sepsis were recorded. Serum albumin levels were recorded on daily basis during the first 48 h after admission. The acute physiology and chronic health evaluation (APACHE) II score, sequential organ failure assessment (SOFA) score, C-reactive protein (CRP), blood lactate, white blood cell (WBC) count, hematocrit, glucose, prothrombin time (PT), total bilirubin, alanine aminotransferase (ALT), procalcitonin (PCT), blood urea nitrogen (BUN), and serum creatinine were also registered once daily. The measurement of IAP was in accordance with the bladder technique recommended by the World Society of Abdominal Compartment Syndrome in 2006 (Cheatham et al., 2007), and the incidence of IAH was also recorded. All of the parameters were counted as the means of two daily values. The number of patients with MODS, repeated surgery and vasopressor administration, and the duration of MV and continuous renal replacement therapy (CRRT) during hospitalization were registered. Moreover, the hospital mortality and duration of ICU stay were also registered.

### Statistical analysis

The Kolmogorov–Smirnov test was initially performed to test normal distribution of the data. Abnormal distributed data were presented as medians (interquartile ranges) and compared by Mann–Whitney U test or Kruskal–Wallis test. Normally distributed data were presented as means ± standard deviation and compared by *t* test or one-way ANOVA. The comparisons of two paired groups were performed by paired *t* test or Wilcoxon signed-rank test. Categorical variables were expressed as absolute numbers or in percentages, and were analyzed using *χ*^2^ test or Fisher’s exact test. To determine the risk factors of hypoalbuminemia, we performed several series of univariate logistics regression analyses using the above-mentioned variables. Variables that showed statistical significance were identified by further multivariate logistic regression analyses with the stepwise method. Receiver operating characteristic (ROC) curves were used to evaluate the ability of every risk factor to predict the development of hypoalbuminemia in sepsis. IBM Statistical Package for the Social Sciences (SPSS, version 20.0, NY, USA) software was used for statistical analysis. *P* < 0.05 was considered statistically significant.

## Results

As shown in Fig. 1, a total of 135 eligible patients with sepsis were included in this clinical retrospective study during the specified period. Of these patients, 45 (33.3%) cases had severe sepsis, and 30 (22.2%) cases had septic shock. The demographic data and clinical parameters of the patients on SICU admission were shown in Table 1. Ninety-five (70.4%) patients developed hypoalbuminemia, and 39 (28.9%) patients died of MODS or septic shock during hospital stay. With respect to the critical complications, 50 (37.0%) patients developed AKI, whereas 44 (32.6%) patients developed ARDS and the same amount of patients developed IAH.

**Table 1:** Demographic data and clinical parameters on admission.

	Group A (*n* = 9)	Group B (*n* = 20)	Group C (*n* = 66)	Group D (*n* = 40)	*P* value
Age (years)	69.0 (67.5–79.5)	69.0 (63.3–73.5)	73.5 (64.5–81.3)	70.5 (60.8–79.5)	0.325
Sex (male: female)	6:3	16:4	39:27	28:12	0.329
Etiology (*n*, %)					
Abdominal infection	8 (88.9%)	17 (85.0%)	44 (66.7%)	25 (62.5%)	0.168
Thoracic/Pulmonary infection	1 (11.1%)	2 (10.0%)	16 (24.2%)	9 (22.5%)	0.479
Urinary infection	–	–	5 (7.6%)	5 (12.5%)	0.498
Mucocutaneous infection	–	–	1 (1.5%)	1 (2.5%)	1.000
Intracranial infection	–	1 (5.0%)	–	–	–
BMI	23.0 (21.8–25.7)	22.0 (20.6–23.9)	23.1 (21.0–25.0)	23.0 (21.9–25.0)	0.365

**Notes.**

Group A albumin level ≤15 g/L Group B 16 ≤ albumin level ≤20 g/L Group C 21 ≤ albumin level ≤25 g/L Group D albumin level ≥26 g/L BMI body mass index

### Risk factors of hypoalbuminemia

Results of the univariate logistic regression analyses of hypoalbuminemia were shown in Table 2. There were significant differences between septic patients with and without hypoalbuminemia with regard to the APACHE II score, CRP, blood lactate, WBC count, PT, PCT, MODS incidence, vasopressor administration, ICU stay, and duration of MV. The further multiple logistic regression analyses of the ten significant variables revealed that three variables were independent risk factors for the development of hypoalbuminemia: APACHE II score, CRP, and blood lactate (shown in Table 3).

**Table 2:** Univariate logistic regression analysis of hypoalbuminemia.

	OR	95% CI	*P* value
APACHE II	1.757	1.419–2.175	<0.001
SOFA	1.102	0.988–1.229	0.080
CRP	1.022	1.013–1.032	<0.001
Blood lactate	1.928	1.406–2.643	<0.001
WBC count	1.109	1.026–1.199	0.009
Hematocrit	1.007	0.966–1.030	0.334
Blood glucose	0.955	0.863–1.057	0.373
PT	1.163	1.063–1.274	0.001
Total bilirubin	0.998	0.987–1.009	0.688
ALT	1.010	0.999–1.021	0.083
PCT	1.098	1.025–1.175	0.007
BUN	0.996	0.955–1.038	0.849
Serum creatinine	1.001	0.998–1.004	0.443
MODS	2.377	1.045–5.410	0.039
IAH	1.672	0.729–3.833	0.224
Repeated surgery	0.968	0.946–1.097	0.325
Vasopressor	1.079	1.018–1.214	0.028
ICU stay	1.090	1.013–1.172	0.021
Duration of CRRT	1.026	0.950–1.108	0.519
Duration of MV	1.182	1.066–1.311	0.002

**Notes.**

APACHE II acute physiology and chronic health evaluation II SOFA sequential organ failure assessment CRP C-reactive protein WBC white blood cell PT prothrombin time ALT alanine aminotransferase PCT procalcitonin BUN blood urea nitrogen MODS multiple organ dysfunction syndrome IAH intra-abdominal hypertension CRRT continuous renal replacement therapy MV mechanical ventilation

**Table 3:** Independent risk factors in multivariate logistic regression analysis of hypoalbuminemia.

	OR	95% CI	*P* value
APACHE II	1.786	1.379–2.314	<0.001
CRP	1.016	1.005–1.027	0.005
Blood lactate	1.764	1.141–2.726	0.011

**Notes.**

APACHE II acute physiology and chronic health evaluation II CRP C-reactive protein

**Figure 2:**
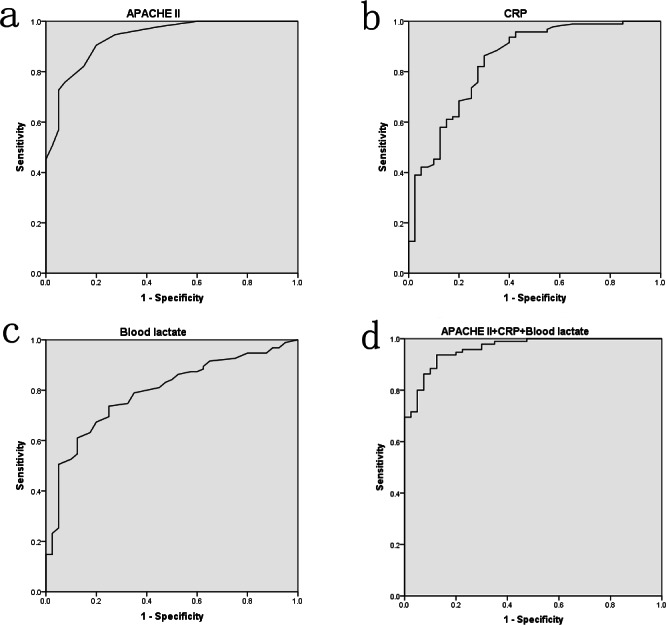
The ROC curve analyses for APACHE II score (A), CRP (B), blood lactate (C) and the combination of three factors (D) on predicting hypoalbuminemia in surgical sepsis.

The ROC curves were performed to assess the value of APACHE II score, CRP, and blood lactate on predicting hypoalbuminemia in sepsis. As Fig. 2 presented, the area under curves (AUCs) of APACHE II score, CRP, and blood lactate were up to 0.933 (*P* < 0.001), 0.842 (*P* < 0.001), and 0.790 (*P* < 0.001), respectively. However, no difference (*P* > 0.05) was found in combination of the three factors though it had a higher AUC value (0.962). Optimal cut-off points for the three factors were also derived from ROC curves. Using cut-off points of 14.5 APACHE II score, 34.25 mg/L CRP, and 2.35 mmol/L blood lactate, the sensitivity of these factors in predicting hypoalbuminemia were respectively 90.5%, 86.3%, and 73.7%, and the corresponding specificity were 80.0%, 70.0%, and 75.0%, respectively.

### Disease severity and outcomes

To investigate the effects of different albumin levels on the disease severity and outcomes of surgical septic patients, we divided patients into four groups according to the levels of albumin. Patients with an albumin level ≤15 g/L were assigned into group A, 16 ≤ albumin level ≤20 g/L into group B, 21 ≤albumin level ≤25 g/L into group C, and albumin level ≥26 g/L into group D.

Table 4 demonstrated the comparisons of severity markers among the four groups. Further subgroup analyses showed that the APACHE II score, CRP, blood lactate, WBC count, PT, ALT, and PCT values of group A were significantly higher than those of the other groups (*P* < 0.05). There were significant differences regarding APACHE II score, CRP, blood lactate, PT, and PCT values between group B and C/D (*P* < 0.05). No differences with regard to all the severity markers except APACHE II score, blood lactate, and PCT values (*P* < 0.05) were observed between group C and D (*P* > 0.05). No differences were observed among four groups (*P* > 0.05) about SOFA score, hematocrit, blood glucose, total bilirubin, BUN, and serum creatinine.

**Table 4:** Clinical markers of disease severity.

	Group A (*n* = 9)	Group B (*n* = 20)	Group C (*n* = 66)	Group D (*n* = 40)	*P* value
APACHE II	27 (25–32)^a^^,^^b^^,^^c^	23 (19.5–26.8)^d^^,^^e^	19 (16–23)^f^	12 (9–14)^g^	<0.001
SOFA	9 (7–10)	6.5 (4–11.5)	8.5 (7–11)	6 (5–9.8)	0.108
CRP (mg/L)	255.3 (220.5–306.0)^a^^,^^b^^,^^c^	111.5 (68.5–187.8)^d^^,^^e^	97.4 (44.8–156.0)	14.7 (8.3–64.3)^g^	<0.001
Blood lactate (mmol/L)	9.6 (7.1–11.8)^a^^,^^b^^,^^c^	4.6 (3.4–6.3)^d^^,^^e^	2.9 (2.0–4.1)^f^	1.7 (1.1–2.5)^g^	<0.001
WBC count (×10^9^/L)	22.0 (18.4–27.9)^a^^,^^b^^,^^c^	13.8 (12.6–17.7)^e^	12.8 (10.4–15.3)	10.9 (8.2–14.0)^g^	<0.001
Hematocrit	0.31(0.25–0.36)	0.29 (0.25–0.36)	0.29 (0.24–0.33)	0.33 (0.29–0.36)	0.069
Blood glucose (mmol/L)	8.5 (6.4–11.5)	8.85 (7.1–10.6)	7.5 (5.2–10.3)	9.3 (7.0–11.1)	0.163
PT (s)	29.0 (23.5–35.2)^a^^,^^b^^,^^c^	20.5 (19.3–23.0)^d^^,^^e^	16.3 (13.9–19.3)	14.1 (13.0–16.1)^g^	<0.001
Total bilirubin (μmol/L)	10.4 (8.2–28.1)	11.1 (4.5–12.8)	13.6 (9.3–19.9)	11.8 (9.2–20.8)	0.071
ALT (U/L)	88.0 (70.5–157.5)^a^^,^^b^^,^^c^	49.0 (37.5–65.0)^d^^,^^e^	23.5 (13.0–46.8)	22.5 (11.0–45.8)^g^	<0.001
PCT (ng/mL)	26.0 (11.8–77.5)^a^^,^^b^^,^^c^	10.1 (4.3–23.9)^d^^,^^e^	4.2 (1.6–10.5)^f^	1.8 (0.5–3.7)^g^	<0.001
BUN (mmol/L)	9.5 (7.2–16.8)	9.9 (7.0–20.3)	14.3 (9.8–19.5)	11.9 (8.2–16.8)	0.331
Serum creatinine (μmol/L)	143.7 (87.7–171.3)	115.1 (60.2–159.9)	159.7 (99.7–232.3)	139.3 (80.8–195.9)	0.353

**Notes.**

CRP C-reactive protein Group A albumin level ≤15 g/L Group B 16 ≤ albumin level ≤20 g/L Group C 21 ≤ albumin level ≤25 g/L Group D albumin level ≥26 g/L APACHE II acute physiology and chronic health evaluation II SOFA sequential organ failure assessment CRP C-reactive protein WBC white blood cell PT prothrombin time ALT alanine aminotransferase PCT procalcitonin BUN blood urea nitrogen

a Significant difference (*P* < 0.05) was found between group A and B.

b Significant difference (*P* < 0.05) was found between group A and C.

c Significant difference (*P* < 0.05) was found between group A and D.

d Significant difference (*P* < 0.05) was found between group B and C.

e Significant difference (*P* < 0.05) was found between group B and D.

f Significant difference (*P* < 0.05) was found between group C and D.

g Significant difference (*P* < 0.05) was found between group (A + B) and (C + D).

Table 5 demonstrated the comparisons of outcome variables among the four groups. Further subgroup analyses showed that the hospital mortality, ICU stay, and MV duration of group A were significantly higher than those of the other groups (*P* < 0.05). And the MODS, IAH, and vasopressor administration incidences of group A were also higher than those of group C/D (*P* < 0.05). The same statistical results of the above six variables were observed between group (A + B) and (C + D) (*P* < 0.05). There were significant differences about hospital mortality (between group B and C only), MV duration (between group B and D only), MODS and vasopressor administration incidences between group B and C/D (*P* < 0.05). No differences regarding all the outcome variables except MV duration (*P* < 0.05) were observed between group C and D (*P* > 0.05).

**Table 5:** Clinical outcome variables.

	Group A (*n* = 9)	Group B (*n* = 20)	Group C (*n* = 66)	Group D (*n* = 40)	*P* value
Hospital mortality (%)	8 (88.9%)^a^^,^^b^^,^^c^	9 (45.0%)^d^	14 (21.2%)	8 (20.0%)^g^	<0.001
MODS (%)	8 (88.9%)^b^^,^^c^	11 (55.0%)^d^^,^^e^	17 (25.8%)	9 (22.5%)^g^	<0.001
IAH (%)	7 (77.8%)^b^^,^^c^	9 (45.0%)	18 (27.3%)	10 (25.0%)^g^	0.009
Repeated surgery (%)	3 (33.3%)	4 (20.0%)	10 (15.2%)	7 (17.5%)	0.600
Vasopressor (%)	7 (77.8%)^b^^,^^c^	8 (40%)^d^^,^^e^	10 (15.2%)	5 (12.5%)^g^	<0.001
ICU stay (days)	16 (10.5–21.5)^a^^,^^b^^,^^c^	6.5 (4.3–12.8)	6 (4–11.3)	5 (3–9.8)^g^	0.006
CRRT (days)	0 (0–0.5)	0 (0–0)	0 (0–0)	0 (0–0)	0.873
MV (days)	12 (9–22)^a^^,^^b^^,^^c^	4.5 (2.5–13.8)^e^	4 (2–7)^f^	1.5 (0.5–5.8)^g^	<0.001

**Notes.**

Group A albumin level ≤15 g/L Group B 16 ≤ albumin level≤20 g/L Group C 21 ≤ albumin level ≤25 g/L Group D albumin level ≥26 g/L MODS multiple organ dysfunction syndrome IAH intra-abdominal hypertension CRRT continuous renal replacement therapy MV mechanical ventilation

a Significant difference (*P* < 0.05) was found between group A and B.

b Significant difference (*P* < 0.05) was found between group A and C.

c Significant difference (*P* < 0.05) was found between group A and D.

d Significant difference (*P* < 0.05) was found between group B and C.

e Significant difference (*P* < 0.05) was found between group B and D.

f Significant difference (*P* < 0.05) was found between group C and D.

g Significant difference (*P* < 0.05) was found between group (A + B) and (C + D).

**Figure 3:**
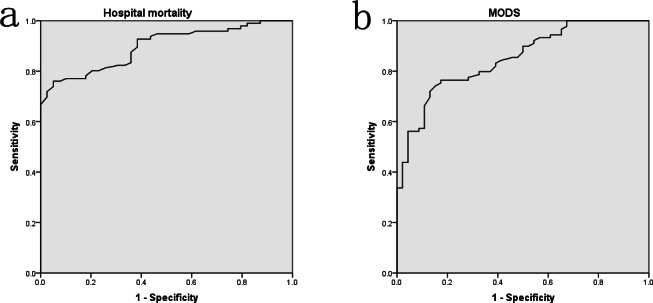
The ROC curve analyses for albumin levels on predicting hospital mortality (A) and MODS incidence (B) in surgical sepsis.

The above results indicated that septic patients with albumin level ≤20 g/L (especially ≤15 g/L) had worst disease severity and outcomes, whereas patients with albumin level between 21 and 25 g/L might have similar prognosis to that of albumin level ≥26 g/L. The ROC curves were also performed to assess the levels of albumin on predicting the prognosis of sepsis. As Fig. 3 presented, the AUCs of hospital mortality and MODS incidence were 0.897 (*P* < 0.001) and 0.851 (*P* < 0.001), respectively. Furthermore, the logistic regression analyses presented that every 1 g/L decrease of albumin level below the optimal cut-off (22.95 g/L) was associated with nearly a 20% increase in hospital mortality (OR 1.194, 95% CI [0.975–1.462], *P* = 0.047). Every 1 g/L decrease of albumin level below the optimal cut-off (22.6 g/L) was associated with nearly a 30% increase in MODS incidence (OR 1.287, 95% CI [1.021–1.623], *P* = 0.034).

## Discussion

Albumin is the main serum protein of human beings, which is synthesized by the liver from amino acids (Gatta, Verardo & Bolognesi, 2012). Normal levels of serum albumin can maintain the colloid osmotic pressure of plasma and prevent the development of tissue edema (Gatta, Verardo & Bolognesi, 2012; Nicholson, Wolmarans & Park, 2000). However, the concentration of serum albumin is often decreased in a great deal of conditions, for example, in major surgery, trauma or infection, and if serum albumin <35 g/L, hypoalbuminemia would be diagnosed. As a severe infection, sepsis is also frequently accompanied by hypoalbuminemia, which is caused mainly by decreased hepatic synthesis, increased leakage into the interstitial compartment, and persistent catabolism (Gatta, Verardo & Bolognesi, 2012; Nicholson, Wolmarans & Park, 2000). Several studies reported that the incidence of hypoalbuminemia (<30–35 g/L) was about 60–80% in critically ill patients (Aguayo-Becerra et al., 2013; Gatta, Verardo & Bolognesi, 2012). Taking into account the clinical significance, we defined hypoalbuminemia as an albumin level of less than 25 g/L, thus its incidence was 70.4% (95/135) in this study, which was also consistent with the previous reports. To the best of our knowledge, this was the first study to investigate the independent risk factors of hypoalbuminemia in surgical septic patients, and three variables (APACHE II score, CRP, and blood lactate) were found to be significantly associated with poor outcome.

The APACHE II score, which included 14 parameters, was considered as an important indicator on assessing disease severity and outcomes of critically ill patients (Sun et al., 2013). Our results revealed that APACHE II score ≥14.5 showed an optimal predictive value for development of hypoalbuminemia, as evidenced by AUC of 0.933 (*P* < 0.001). However, the mechanisms underlying this correlation were complicated and unclear. We speculated that patients with higher APACHE II score might have more severe infection, more aggravated capillary leak, and then suffer more rapid albumin reduction (Gatta, Verardo & Bolognesi, 2012; Nicholson, Wolmarans & Park, 2000). However, the main limitation of APACHE II score was its complexity and the ideal predictors of hypoalbuminemia would be a single factor or a combination of several simple factors. Of the three predictors established, CRP and blood lactate conformed to these requirements. CRP also demonstrated a convincing predictive value of hypoalbuminemia, as evidenced by AUC of 0.842 (*P* < 0.001), and its optimal cut-off point was 34.25 mg/L. As a positive acute-phase protein, CRP would be increased obviously in the conditions of acute trauma, inflammation or infection. These acute stress responses could decrease the rate of albumin mRNA transcription and the synthesis of albumin (Nicholson, Wolmarans & Park, 2000). Therefore, as a negative acute-phase protein, albumin might be negatively correlated with CRP during the acute phase of critical illness. Our results about CRP were in accordance with the above-mentioned findings. In addition, the present study also found that blood lactate ≥2.35 mmol/L demonstrated an optimal predictive value for development of hypoalbuminemia, as evidenced by AUC of 0.790 (*P* < 0.001). In sepsis, tissue hypoxia or visceral hypoperfusion induced by hypovolemia, hypotension or microcirculatory dysfunction were considered as the predominant causes of hyperlactatemia (Garcia-Alvarez, Marik & Bellomo, 2014; Thomas-Rueddel et al., 2014). Sustained hyperlactatemia could lead to hepatic dysfunction (Thomas-Rueddel et al., 2014) and then impair the synthesis and secretion of albumin. On the other hand, the emergence of hyperlactatemia also illustrated an obvious microcirculatory dysfunction and increased capillary permeability of sepsis, which were confirmed to be the predominant pathophysiology mechanisms of hypoalbuminemia (Gatta, Verardo & Bolognesi, 2012). Therefore, blood lactate might be also negatively correlated with albumin during the acute phase of sepsis.

Some literatures reported that albumin levels were associated with the prognosis of critically ill patients (Aguayo-Becerra et al., 2013; Chou et al., 2009). Aguayo-Becerra et al. (2013) divided albumin levels into three groups: >30 g/L, 20–30 g/L, and <20 g/L in burn patients, and found that patients with albumin levels <20 g/L had a higher mortality risk, with 84% sensitivity and 83% specificity. Serra et al. (2014) found that low serum albumin level was an independent determinant of pressure ulcer occurrence in ICU patients. However, few studies investigated the effects of different albumin levels on the prognosis of patients with surgical sepsis. The findings of our study showed that patients with albumin level ≤15 g/L had the worst disease severity and outcomes, and patients with albumin level ≤20 g/L also had worse prognosis than that of albumin level ≥21 g/L, whereas patients with albumin level between 21 and 25 g/L had a nearly identical outcome with that of albumin level ≥26 g/L. The AUCs of ROC curves also indicated that albumin levels had satisfactory predictive value on MODS (AUC 0.851) and hospital mortality (AUC 0.897). Moreover, the logistic regression analyses presented that albumin levels were negatively correlated with MODS incidence and hospital mortality when below the optimal cut-off values (22.6 and 22.95 g/L, respectively). These results revealed that albumin levels were negatively correlated the prognosis of surgical sepsis when below about 23 g/L.

Now that hypoalbuminemia was significantly associated with the prognosis of sepsis, researchers were trying to infuse human albumin solutions to improve the outcomes of septic patients. Unfortunately, the conclusions were controversial and neither efficacy nor safety of human albumin was established. In most cases, albumin administration seemed to be used mainly for resuscitation and volume expansion rather than correcting hypoalbuminemia. Delaney et al. (2011) performed a meta-analysis reporting that resuscitation with albumin-containing solutions was associated with lower mortality in patients with sepsis, whereas the SAFE Study Investigators (Finfer et al., 2004) and the ALBIOS Study Investigators (Caironi et al., 2014) both confirmed that albumin replacement did not improve the outcomes of critical ill patients. In addition, the subgroup analyses of ALBIOS Study showed that the patients with an albumin concentration of 30 g/L or more did not have a higher survival rate at 28 and 90 days. In this study, we found that patients with albumin level between 21 and 25 g/L had a nearly identical outcome with that of albumin level ≥26 g/L (including ≥30 g/L), while patients with albumin level ≤20 g/L had worse prognosis than that of albumin level ≥21 g/L. Not coincidentally, Chou et al. (2009) determined that albumin administration might reduce the 28-day mortality of severe septic patients with serum albumin ≤20 g/L, but no such effect was found in patients with albumin >20 g/L.

Several limitations of our study should be discussed. Due to the small sample size and the single-center design, our results might be uncertain for a definite conclusion, and the accuracy should be tested by further large-scale studies. Moreover, the number of patients receiving albumin infusion and the volume of albumin solutions were not recorded in this study, which might affect the prognostic analysis of patients with hypoalbuminemia. Finally, because our parameters were recorded only during the first 48 h after ICU admission, the risk factors and prognostic value of hypoalbuminemia in the late stage of sepsis also requires validating through future studies.

## Conclusions

This study established that APACHE II score (≥14.5), CRP (≥34.25 mg/L), and blood lactate (≥2.35 mmol/L) were the independent risk factors of hypoalbuminemia in the early stage of surgical sepsis. Patients with baseline albumin level ≤20 g/L had worse prognosis than that of albumin level ≥21 g/L. Albumin levels were negatively correlated the prognosis of surgical sepsis when below about 23 g/L.

## Supplemental Information

10.7717/peerj.1267/supp-1 Figure S1 Figure 1

10.7717/peerj.1267/supp-2 Figure S2 The ROC curve analyses for APACHE II score (A), CRP (B), blood lactate (C) and the combination of three factors (D) on predicting hypoalbuminemia in surgical sepsis

10.7717/peerj.1267/supp-3 Figure S3 The ROC curve analyses for albumin levels on predicting h ospital mortality (A) and MODS incidence (B) in surgical sepsis

10.7717/peerj.1267/supp-4 Supplemental Information 1 Raw data 1

10.7717/peerj.1267/supp-5 Supplemental Information 2 Raw data 2
