# Supplementary figures and images for: Risk factors and prognosis of hypoalbuminemia in surgical septic patients
Source: PeerJ. 2015 Oct 1;3:e1267. doi: 10.7717/peerj.1267 (PMC4636415; doi:10.7717/peerj.1267)

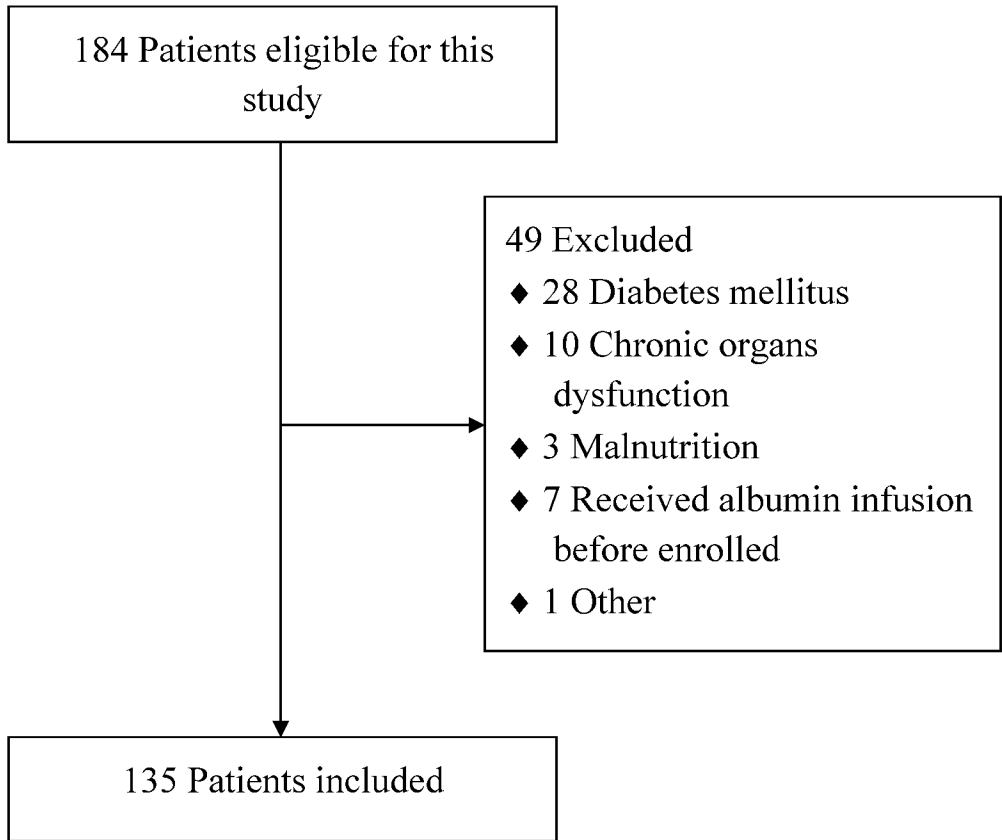

Supplement: Figure S1 [file peerj-03-1267-s001.pdf]

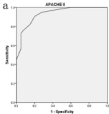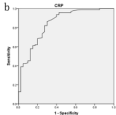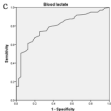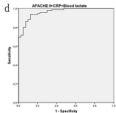

Supplement: Figure S2 [file peerj-03-1267-s002.pdf]

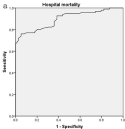

Diagonal segments are produced by ties.

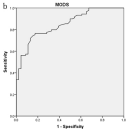

Diagonal segments are produced by ties.

Supplement: Figure S3 [file peerj-03-1267-s003.pdf]
